# Nano-metal–organic frameworks as corrosion inhibitors for strengthening anti-corrosion behavior of carbon steel in a sulfuric acid environment: from synthesis to applications†

**DOI:** 10.1039/d3ra01644g

**Published:** 2023-05-18

**Authors:** S. E. H. Etaiw, G. S. Hassan, A. A. El-Hossiany, A. S. Fouda

**Affiliations:** a Department of Chemistry, Faculty of Science, Tanta University Tanta 31527 Egypt; b Department of Chemistry, Faculty of Science, Mansoura University Mansoura 35516 Egypt asfouda@hotmail.com +20 50 2202264 +20 50 2365730; c Delta for Fertilizers and Chemical Industries Talkha Egypt

## Abstract

In order to demonstrate the effect of Nano-metal organic frameworks, [Cu_2_(CN)_4_(Ph_3_Sn) (Pyz2-caH)_2_] (NMOF1) and [_∞_^3^[Cu(CN)_2_(Me_3_Sn)(Pyz)]] (NMOF2) as corrosion inhibitors for C-steel in 0.5 M sulfuric acid solutions, the following methods were utilized: mass reduction (MR), potentiodynamic polarization (PDP), and AC electrochemical impedance (EIS). The results of the experiments showed that by increasing the dose of these compounds, the inhibition efficacy (*η*%) of C-steel corrosion increased and reached 74.4–90% for NMOF2 and NMOF1 at a dose of 25 × 10^−6^ M, respectively. On the other hand, the *η*% decreased as the temperature range rose. Parameters for activation and adsorption were determined and discussed. Both NMOF2 and NMOF1 were physically adsorbed on the surface of C-steel and conformed to the Langmuir adsorption isotherm model. The PDP studies revealed that these compounds functioned as mixed type inhibitors, *i.e.* affecting both metal dissolution and hydrogen evolution reactions. Attenuated Total Reflection Infra-Red (ATR-IR) analysis was carried out to determine the morphology of the inhibited C-steel surface. There is good agreement between the findings of EIS, PDP and MR.

## Abstract 2

Nano-metal organic frameworks were used as corrosion inhibitors for C-steel in 0.5 M H_2_SO_4_.

## Graphical abstract

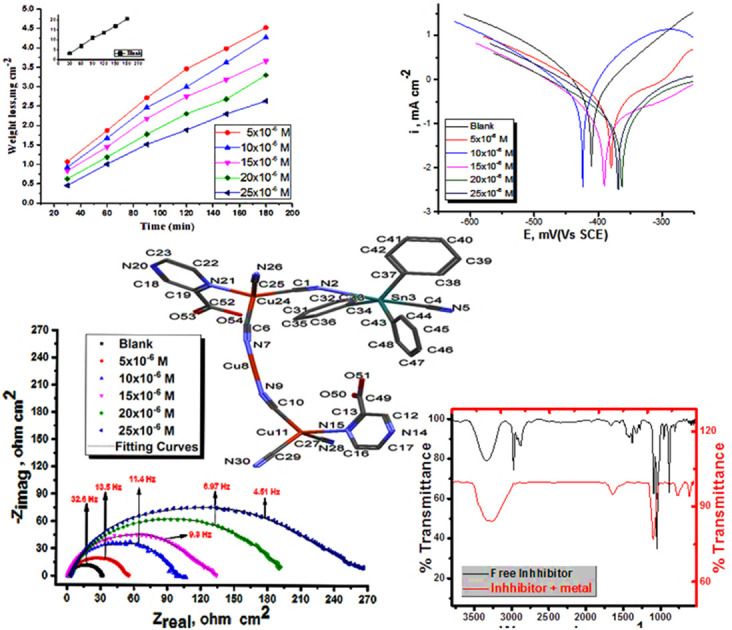

## 1. Introduction

Steel corrosion is a significant industrial problem that affects the entire world and causes serious environmental problems as well as significant financial losses, material failure, and decreased efficiency. In many industrial procedures, including pickling, oil well acidification, cleaning, decalcification, and petrochemical processes, acid solutions are widely used. As a result, metals and alloys corrode more quickly in these extreme acidic conditions. Therefore, the protection of metals from corrosion is of great importance and has been receiving a considerable amount of attention over the past few decades.^1–7^ Numerous industries have conducted extensive study on corrosion inhibitors to slow down how quickly metal dissolves when it meets corrosive surroundings.^8–12^ Corrosion inhibitors with high efficacy have been demonstrated to be associated with their ability to absorb on metal surfaces.^13–15^

Studies indicate that the physicochemical characteristics of the molecule related to its functional group, possible steric effects, and electronic density of donor atoms play a significant role in the adsorption of organic inhibitors.

Adsorption is also thought to be influenced by the potential relationship of the inhibitor's-orbital with the surface atoms d-orbital. A corrosion-protective layer developed on the surface of C-steel as a result of enhanced inhibitor adsorption.^16^

The authors focused on using readily available, inexpensive, environmentally friendly, and renewable sources of organic compounds as inhibitors because these compounds have a strong affinity to prevent metal corrosion in acidic solutions due to the presence of heteroatoms like O, N, and S in their molecular structure.^17–21^ Previous research has demonstrated that organic compounds with heteroatoms like N, O, S, and others, as well as aromatic rings, function as effective corrosion-preventing agents.

The main components of MOFs, which are a new kind of porous crystalline organic–inorganic hybrid material, are metal cations and organic ligands. The effectiveness of the sacrifice is increased by including additional electropositive metals in the organic framework. On the other hand, the organic structure creates a shield over the metal surface that prevents corrosion. Three-dimensional networks of silver-based MOFs that were suitable for preventing C-steel corrosion in 1 M HCl solution were described in a few literature-based research,^22^ proving that MOF may be used as a powerful corrosion inhibitor.

In a different study, metal organic frameworks with both silver and nitrogen donors were demonstrated to be effective inhibitors of Cu corrosion in HCl solution. As corrosion inhibitors for metals and alloys, MOFs with organic ligands that are substituted aryl, heteroaryl, or heterocyclic compounds containing an exocyclic sulfur group have also been characterized.^23,24^

The literature had reported the discovery of a novel MOF from Cd.^25^ In 2017, a report on the effects of Co, Ni, and Cu metal-based MOFs on the prevention of mild steel corrosion was published.^26^ ZIF-8 and other hydrophobic MOFs are used in the anticorrosion industry as a consequence of MOF research.^27,28^ Recent research developed an anticorrosive coating for the petrochemical industry utilizing samarium(iii) nitrate and [bis(phosphonomethyl)amino] methyl phosphonic acid (ATMP).^29^ According to thermodynamic research,^30^ the extremely excellent inhibitory property of MOF on metal surface was attributed to chemisorption of MOF on metal surface. Silver(i) pyrazine complex^31^ and silver(i) quinoxaline metal organic framework (MOF)^32^ were recently tested as corrosion inhibitors. Etaiw *et al.*^33^ utilized MOF [(AgCN)_4_-(qox)_2_], as corrosion inhibitor for carbon steel in 1 M HCl solution. The inhibition efficacy was reached to 89.3% at 1 × 10^−4^ M. Izuchukwu *et al.*^34^ synthesized ZnMOF-BTA framework and used it as corrosion inhibitor for Q235 C-steel of two types of MOFs, namely Mg(C7)_2_ and Mg(C10)_2_, for magnesium corrosion inhibition in a neutral pH solution. They concluded that Mg(C10)_2_ framework reveals better corrosion inhibition efficiency due to its lower solubility compared to Mg(C7)_2_ structures, although both structures are very similar. A new metal–organic framework [Ag(qox)(4-ab)] was used as a corrosion inhibitor for carbon steel in HCl solution.^35^ Khaled *et al.*^36^ synthesized two new metal–organic frameworks based on silver(i) and nitrogen donors to investigate copper corrosion inhibition in HCl solution. They found out that the adsorption of both MOFs is physically obeyed by Langmuir adsorption isotherm. Furthermore, they studied the effect of temperature as one of the most significant parameters affecting corrosion efficiency. Finally, they concluded that the MOF synthesized with quinoxalinecarboxylic acid shows better efficiency in comparison to those containing pyrazine carboxylic acid. Etaiw *et al.*^37^ synthesized a new metal–organic framework based on cadmium thiocyanate to investigate the corrosion inhibition of copper in HCl solution. Their results showed that the adsorption of the as-synthesized MOF was based on Langmuir adsorption isotherm. Furthermore, only the effect of temperature on the corrosion inhibition efficiency was investigated. ZIF-8 and ZIF-8@{Mo132}were synthesized and utilized as corrosion inhibitors^38^ for C-steel in 1 M HCl solution. The inhibitor efficacy of ZIF-8@{Mo132} nano-structure (92.3% at 700 ppm) is always higher than that of pristine ZIF-8 (87.4% at 700 ppm).

The following factors informed the selection of NMOFs 1 & 2 for the current investigation: In addition to being easily produced from elements that are very inexpensive, it also has two heterocyclic moieties, four or two cyanide groups, and aromatic systems with N atoms that can enhance the adsorption of inhibitor molecules onto the surface of C-steel.

NMOFs 1 & 2 are supramolecular in size compared to most heterocyclic compounds. MOFs have significant advantages such as: (i) uniform structures, ultrahigh porosity, tunable composition, and easy-to-functionalize surface, due to many applications of MOFs in many fields as anticorrosion coatings and (ii) the use of organic–inorganic hybrid [metal–organic frameworks (MOFs)] inhibitors, especially for carbon steel protection, has been less reported so far. So, we examined the use of NMOF1 and NMOF2 as corrosive inhibitors for C-steel in 0.5 M H_2_SO_4_ solutions using MR method and the electrochemical techniques (PDP, EIS).

The morphology of the tested NMOFs had been investigated by transmission electron microscopy (TEM) measurements. Also, the analysis of C-steel surface was evaluated using attenuated total reflection infrared (ATR-IR) technique.

## 2. Experimental

### 2.1. Composition of C-steel samples

The experiments were performed with C-steel type C1018 with the composition as in Table 1.

**Table 1:** C-steel chemical composition

Elements	C	Mn	P	Si	Fe
Weight%	0.20%	0.60%	0.004%	0.003%	Rest

For MR measurements, rectangular specimens with dimensions of 2 × 2 × 0.2 cm were utilized. The exposed surface area of C-steel for electrochemical testing was 1 cm^2^.

### 2.2. Chemicals

1 × 10^−3^ M stock solution of NMOFs was diluted *via* deionized H_2_O to obtain different inhibitor concentrations (5–25 × 10^−6^ M). In 0.5 M H_2_SO_4_, the maximal dose of a metal–organic compound was reported to be 25 × 10^−6^ M. The metal–organic compound employed in this work is highly soluble in water, has higher molecular weights, and includes a significant number of donating atoms (N and O) and easily available, non-toxic and their structures are listed in Table 2.

**Table 2:** Chemical structure of the nano metal–organic frameworks (NMOF1 & NMOF2)

Compound	NMOF1	NMOF2
Structure	[Cu_2_(CN)_4_(Ph_3_Sn)·(Pyz2-caH)_2_]	[_∞_^3^[Cu(CN)_2_(Me_3_Sn)(Pyz)]]
Mol. Wt	MW = 829.36 g mol^−1^	MW = 359.46 g mol^−1^
Mol. formula	C_32_H_23_N_8_O_4_Cu_2_Sn	C_9_H_13_N_4_CuSn

#### 2.2.1. Synthesis of NMOF1 & NMOF2

##### 2.2.1.1. Preparation of [Cu_2_(CN)_3_(Ph_3_Sn)·(Pyz2-caH)_2_], (NMOF1)

45 mg of K_3_ [Cu(CN)_4_] (0.157 mmol) was dissolved in 10 mL H_2_O, under stirring to the solutions of 0.385 mg Ph_3_SnCl (1.0 mmol) in 10 mL acetonitrile and 0.0620 mg Pyz2-caH (0.50 mmol) in 10 mL acetonitrile. The mixture of solutions was ultrasonically irradiated for 2 h with a distinct power of 70 W at 28 °C. Already, after several days, nano-yellow precipitate of NMOF1 started growing from the initially clear solution.^39^ Anal. calcd. for NMOF1: (%): C, 46.32; H, 2.77; N, 13.49; Cu, 15.66. Found: C, 46.08; H, 2.69; N, 13.39; Cu, 15.56.

##### 2.2.1.2. Synthesis of [_∞_^3^[Cu(CN)_2_(Me_3_Sn)(Pyz)]], (NMOF2)

Nano sized NMOF2 had been prepared using a solution of 180 mg (0.65 mmol) of K_3_ [Cu(CN)_4_] in 10 mL of water to a solution of 73 mg (0.63 mmol) of Pyz in 10 mL of acetonitrile placed in an ultrasonic bath. To this solution was added dropwise an aqueous solution of 380 mg (1.9 mmol) of Me_3_SnCl. The obtained mixture of solutions was ultrasonically irradiated for 60 min with a distinct power of 70 W at 30 °C. After that, the resulting precipitate was isolated by centrifugation, washed with 20 mL of water followed by acetonitrile and dried in open air. Nanoparticles of NMOF2 were collected in a yield of about 89%.^39^ Anal. calcd for NMOF2 (%): C, 30.07; H, 3.65; N, 15.59; Cu, 17.68; found (%): C, 30.41; H, 3.91; N, 14.52; Cu, 17.79. Single-crystal X-ray diffraction measurements were carried out using a Kappa CCD Enraf Nonius FR 90 four-circle goniometer with graphite monochromatic Mo Kα radiation (*λ* = 0.71073 Å) at 25 ± 2 °C. Structure solution, refinement by Full-matrix least-squares techniques on *F*^2^ and data output were completed using SHELX-97 and SIR92 programs.^40^

### 2.3. Methods

#### 2.3.1. Mass reduction (MR) tests

The usual technique for measuring the dissolution rate and inhibition efficacy (percent IE) is MR approach in which a 2 × 2 × 0.2 cm^2^ piece of metal is used. The samples are cut and sanded as previously, then washed with double distilled water, dried, and weighed before being placed in solutions made from varying dosages of metal–organic compounds ranging from 5 × 10^−6^ to 5 × 10^−6^ M in a beaker containing 0.5 M H_2_SO_4_ and changing quantities of metal–organic compound inhibitors for 3 hours, and metal–organic compound inhibitors in varying concentrations for 3 hours. This happens in the presence of 0.5 M H_2_SO_4_ when compared to a sample put in a solution of 0.5 M sulfuric acid without the addition of metal–organic compounds. The samples are weighed before being re-immersed in respective solutions. The temperature varies between 298 and 318 K. After drying thoroughly, for 3 hours, it was put in a beaker with 0.5 M H_2_SO_4_ and varying amounts of metal–organic inhibitors. All experiments were repeated three times for reproducibility.

#### 2.3.2. Electrochemical tests

##### 2.3.2.1. Tests of PDP

The capacity of PDP was adjusted automatically from −700 to +700 mV against (*E*_ocp_), at a scan rate of 0.2 mV s^−1^.

##### 2.3.2.2. Tests of EIS

All open-circuit testing with EIS were carried out with AC signals ranging from 100 kHz to 0.1 Hz and peak amplitudes of 10 mV at open circuit potential (OCP). The equipment used in electrochemical experiments was a Gamry Potentiostat/Galvanostat/ZRA (PCI4-G750). Gamry comprises the DC105 DC Corrosion Program, the EIS300 EIS Program, and a data gathering computer. To plot and compute data, Echem Analyst version 5.5 was used.

### 2.4. Morphology of the surface

#### 2.4.1. Attenuated Total Reflection Infra-Red (ATR-IR) analysis

ATR-IR spectra were recorded in the spectral region 4000 to 500 cm using the Attenuated Total Reflectance (ATR) technique on an FTIR-Spectrometer iS 10 (Thermo Fisher Scientific, USA). The FT-IR spectrum is a useful tool for comparing inhibitor and corrosion products following inhibitor adsorption. After immersion for a period, the FT-IR peak values for metal–organic and C-steel were obtained. After 24 hours of immersion in the acid corrosive solution with 25 × 10^−6^ M of metal–organic, the peak values of the FT-IR were recorded for metal–organic and C-steel.^41^

## 3. Results data and discussion

TEM images of NMOFs (1 and 2) support the nano scale of the particles ranging from 14.22 to 30.84 nm for NMOF1 and 19.25–41.65 nm for NMOF2, Fig. SF1.† The morphology shows regular nanostructures with spherical shapes.

### 3.1. Structure of [Cu_2_(CN)_3_(Ph_3_Sn)(Pyz2-caH)_2_], NMOF1

**Fig. 1:**
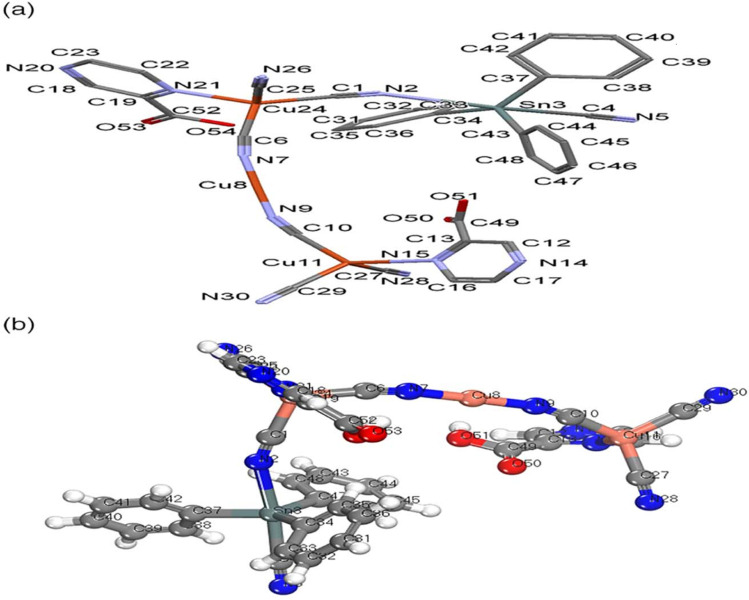
NMOF1 structure (a) mercury, with atom labeling scheme, hydrogen atoms are omitted for clarity (b) bmp structure.

The structure of NMOF1 was confirmed using analytical data and elemental analysis that are compatible to its structure based on computational methods. The magnetic moment of NMOF1 indicates diamagnetic of the NMOF1 and the presence of Cu(i). The molar conductance data support the nonelectrolyte nature having Λ*m* = 9.6 ohm^−1^ cm^2^ mol^−1^, respectively. Molecular structure along with atom numbering of NMOF1 is shown in Fig. 1a and b. Analysis of the bond lengths and bond angles are listed in Table SF1.† The Cu^1^ atoms (Cu11 and Cu25) acquire slightly distorted tetrahedral (T-4) geometry where they coordinate to three ordered cyanide groups and one nitrogen atom of Pyz2-caH ligand. The (Cu8) atom exhibits linear geometry with coordination environment containing two ordered cyanide ligands. The Sn atom coordinates to three phenyl groups and two cyanide ligands creating TBPY-5 configuration, Fig. 1. The Cu–CN bond angles exhibit slight bent structures which are more pronounced in case of Cu25 and Sn atoms (165.370°), Table SF1.† The bond lengths and bond angles are in the normal range reported for the proto-type compounds.^42^ The cyanide ligand acquires different bonds where two cyanide ligands connect one Cu atom (1.121–1021 Å), which was confirmed by the appearance of two *ν*_CN_ IR-bands at 2120 and 2102 cm^−1^.

### 3.2. Structural description of [_∞_^3^[Cu(CN)_2_(Me_3_Sn)(Pyz)]] NMOF2

**Fig. 2:**
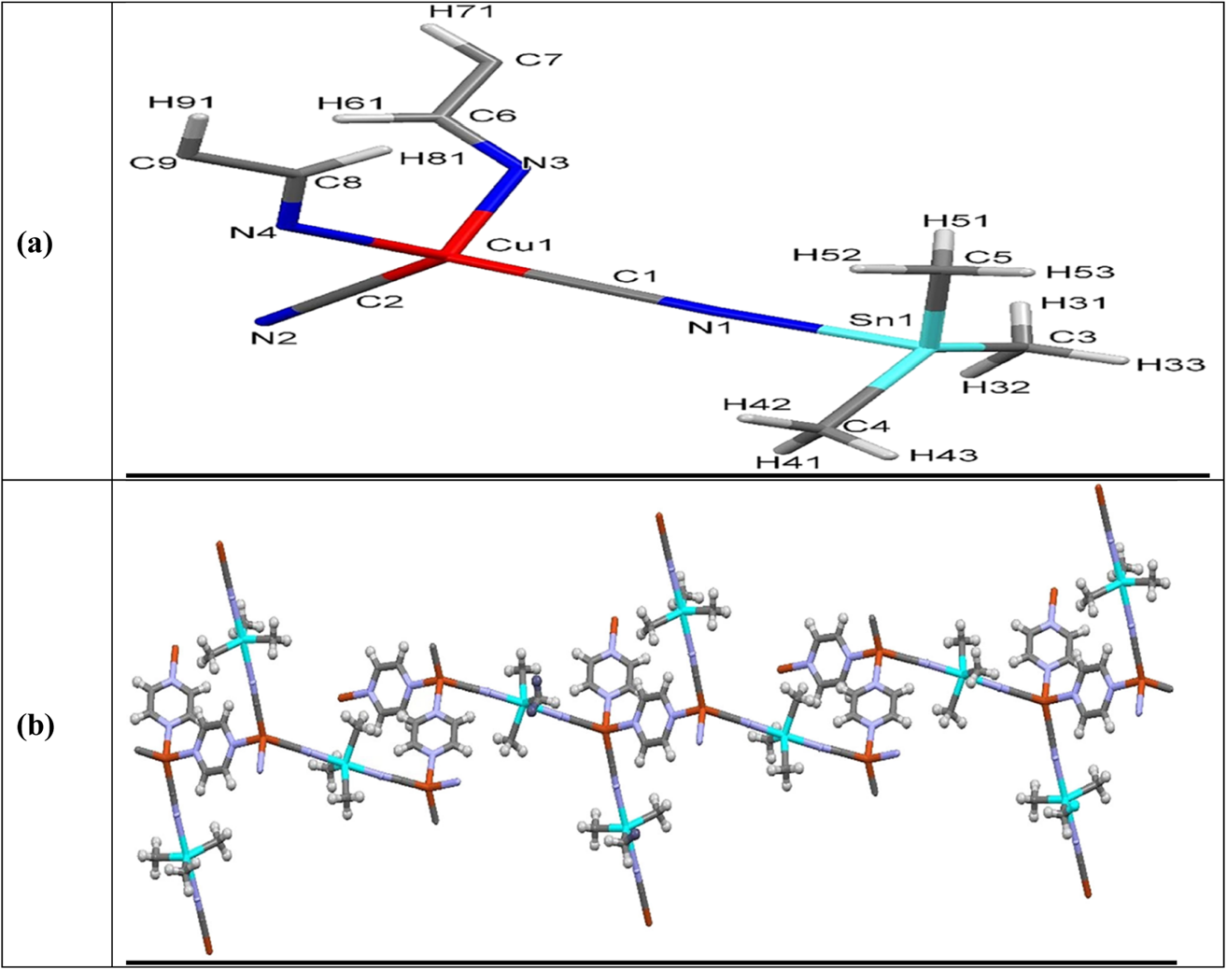
Asymmetric unit of NMOF2 (a) along *a*-axis showing atom labeling scheme (b) 1D-chain of MOF2 in *ab*-plane.

Direct synthesis of NMOF2 by the addition of Pyz, CuCN and Me_3_SnCl at ambient conditions was not verified due to the insolubility of CuCN in most common solvents. On the other hand, addition of CuCN with excess KCN, Me_3_SnCl and Pyz exclusively gave orange crystals of MOF2 at ambient conditions. NMOF2 structure exhibits the molecular formula [C_9_H_13_N_4_CuSn] which had been confirmed by elemental analyses meanwhile its chemical composition of [^3^_∞_[Cu(CN)_2_(Me_3_Sn)(Pyz)]]≡[^3^_∞_[Cu(CN)_2_μ-(Me_3_Sn)μ-(Pyz)]] was elucidated by single-crystal X-ray study. The intermolecular bond lengths and bond angles are collected in Table SF2.† Asymmetric unit of 2 contains one Me_3_Sn, one copper atom, 2 cyanide groups and one Pyz ligand meanwhile two asymmetric units represent the unit cell structure which are bridged *via* Pyrazine, Fig. 2a and b. The Cu(i) atom forms tetrahedral structure by linking to 2 cyanide groups and 2 Pyz ligands *via* nitrogen atoms. Bond lengths and bond angles of the Cu(i) site equal to 1.9207(13)–2.1359(11) Å and 102.47(5)–113.02(5)°, respectively, supporting tetrahedral geometry, Table SF2.† The tin atom acquires trigonal bipyramid geometry *via* coordination to 3 methyl ligands and 2 cyanide groups. The Sn–N and Sn–C bond lengths and angles are in the normal reported range^43^ (Table SF2†). The Cu–CN and Sn-NC fragments adopt nearly linear configuration (Table SF2†).

### 3.3. Mass reduction (MR) tests

The mass loss (Δ*W*) which calculated from MR is given by eqn (1):

1 Δ*W* = (*W*_1_ – *W*_2_)/*a*

*W*_1_, *W*_2_ are the weights of the C-steel specimens before and after reaction with solution and *a* is the area of the specimens in cm^2^.

**Fig. 3:**
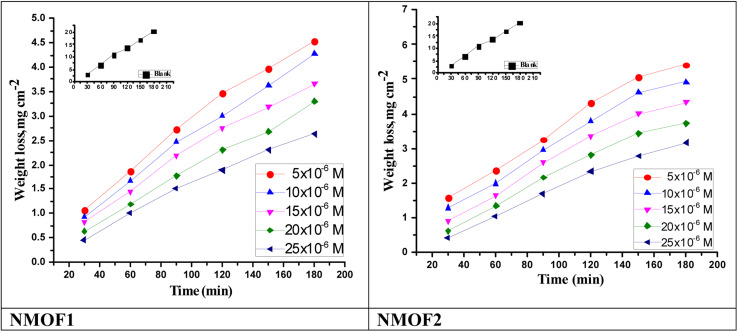
Time–MR curves of C-steel in sulfuric acid solution and in attendance of altered doses of NMOF1 & NMOF2 at 298 K.

Eqn (2) was used to calculate the IE percentage:

2 *η*% = (Δ*W* − Δ*W*_i_)/Δ*W* × 100

where Δ*W* and Δ*W*_i_ represent the MR per unit area in the absence and presence of prepared samples, respectively. This measurement was performed in accordance with ASTM standard G 31-72.^44^ The MR–time curves for C-steel in the presence and absence of changed dosages ranging from 5 × 10^−6^ to 25 × 10^−6^ M for NMOF1 and NMOF2 are shown in Fig. 3. The *k*_corr_ grew as the temperature increased, therefore the *k*_corr_ increased while the IE percent decreased. The curves in the presence of inhibitors are lower than those in the absence of inhibitors. The higher *η*% with increased dosage of metal–organic compounds can be attributed to the formation of an inhibitor layer on the C-steel surface *via* adsorption. This layer is formed by the free electron pairs on the oxygen and nitrogen atoms of metal–organic compound molecules, as well as the π-electrons of aromatic rings. The reduction in *η*% with growing temperature is most likely due to a higher rate of desorption, which is physical adsorption; the *η*% order was: NMOF1 > NMOF2. Table 3 for example, shows the *η*% and *k*_corr_ at various doses of metal–organic NMOF1 of C-steel at temperatures ranging from 298 to 318 K for 120 minutes immersion. As seen in Table 3, raising the temperature lowers the *η*% while raising the inhibitor doses raises it.

**Table 3:** (*η*%) at various dosages of NMOFs 1 and 2 of C-steel for 120 minutes immersion at 298–318 K temperature range

Temp., °C	Conc., M	NMOF1		NMOF2	
		*k*_corr_ (mg cm^−2^ min^−1^)	*η*%	*k*_corr_ (mg cm^−2^ min^−1^)	*η*%
25	**Blank**	**0.10689** ± 0.0017	—	**0.10689** ± 0.0021	**—**
	5 × 10^−6^	0.02882 ± 0.0020	74.4	0.03553 ± 0.0023	66.8
	10 × 10^−6^	0.02555 ± 0.0019	77.3	0.02806 ± 0.0011	73.7
	15 × 10^−6^	0.02065 ± 0.0025	79.7	0.02142 ± 0.0013	80.0
	20 × 10^−6^	0.01652 ± 0.0014	83.1	0.01757 ± 0.0015	83.6
	25 × 10^−6^	0.00955 ± 0.0022	90.0	0.01289 ± 0.0017	87.9
30	**Blank**	**0.13398** ± 0.0023	**—**	**0.13398** ± 0.0020	**—**
	5 × 10^−6^	0.04073 ± 0.0020	69.6	0.050778 ± 0.0023	62.1
	10 × 10^−6^	0.036577 ± 0.0015	72.7	0.040462 ± 0.0014	69.8
	15 × 10^−6^	0.032423 ± 0.0018	75.8	0.032959 ± 0.0019	75.4
	20 × 10^−6^	0.02693 ± 0.0018	79.9	0.0276 ± 0.0018	79.4
	25 × 10^−6^	0.01942 ± 0.0021	85.5	0.023179 ± 0.0016	82.7
35	**Blank**	**0.19856** ± 0.0016	**—**	**0.19856** ± 0.0023	**—**
	5 × 10^−6^	0.06592 ± 0.0019	66.8	0.081608 ± 0.0021	58.9
	10 × 10^−6^	0.06036 ± 0.0015	69.6	0.064731 ± 0.0016	67.4
	15 × 10^−6^	0.05261 ± 0.0019	73.5	0.058575 ± 0.0015	70.5
	20 × 10^−6^	0.0413 ± 0.0025	79.2	0.050236 ± 0.0014	74.7
	25 × 10^−6^	0.03454 ± 0.0013	82.6	0.041698 ± 0.0015	79.0
40	**Blank**	**0.26488** ± 0.0020	**—**	**0.26488** ± 0.0018	**—**
	5 × 10^−6^	0.105422 ± 0.0012	60.2	0.13191 ± 0.0021	50.2
	10 × 10^−6^	0.100 654 ± 0.0015	62.0	0.127 407 ± 0.0018	51.9
	15 × 10^−6^	0.090589 ± 0.0023	65.8	0.094562 ± 0.0024	64.3
	20 × 10^−6^	0.076285 ± 0.0021	71.2	0.084497 ± 0.0022	68.1
	25 × 10^−6^	0.061982 ± 0.0015	76.6	0.07761 ± 0.0020	70.7
45	**Blank**	**0.31742** ± 0.0017	**—**	**0.31742** ± 0.0021	**—**
	5 × 10^−6^	0.144109 ± 0.0002	54.6	0.181247 ± 0.0015	42.9
	10 × 10^−6^	0.132047 ± 0.0021	58.4	0.166646 ± 0.0019	47.5
	15 × 10^−6^	0.119667 ± 0.0024	62.3	0.130777 ± 0.0017	58.8
	20 × 10^−6^	0.092687 ± 0.0017	70.8	0.113954 ± 0.0014	64.1
	25 × 10^−6^	0.078403 ± 0.0016	75.3	0.097131 ± 0.0013	69.4

#### 3.3.1. Temperature influence on corrosion procedure

**Fig. 4:**
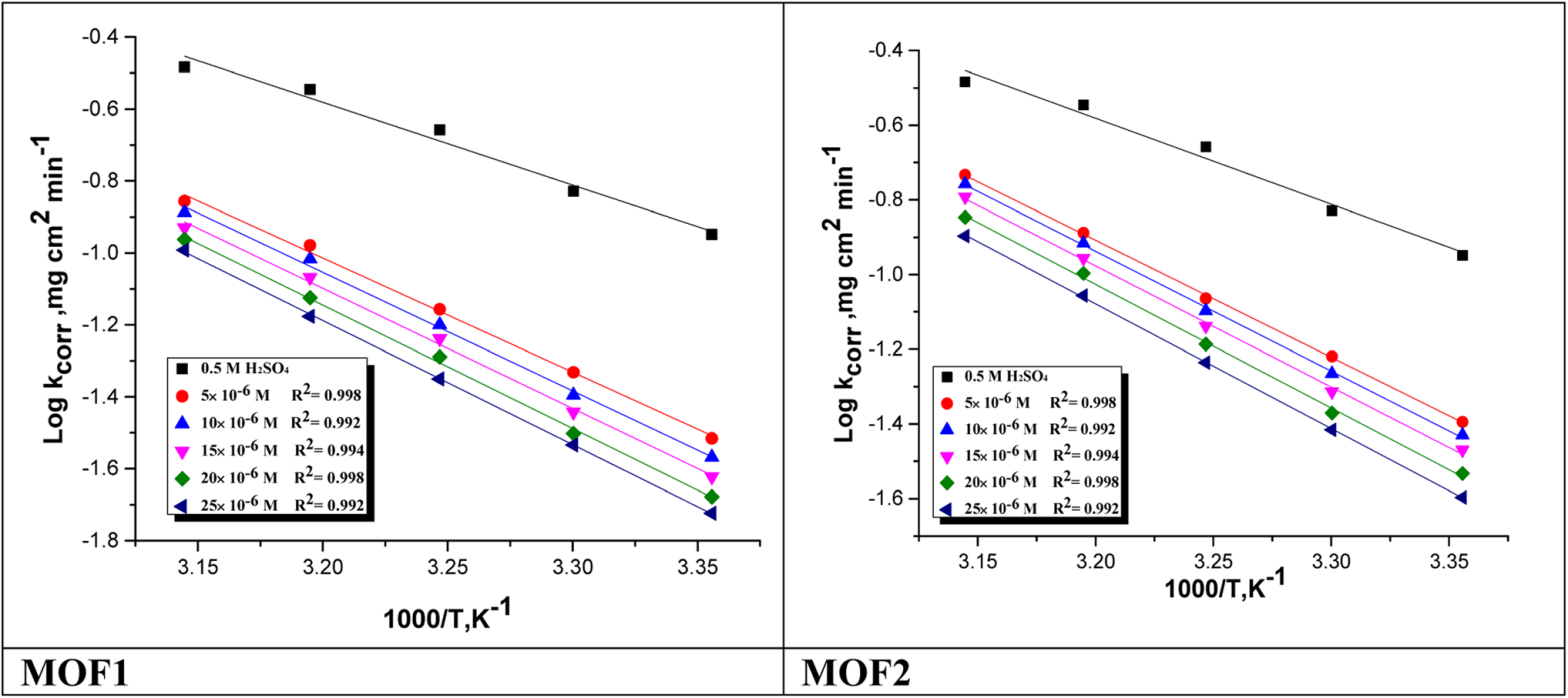
log *k*_corr_*vs.* 1/*T* of the investigated NMOF1 & NMOF2 with and without altered doses of investigated compounds at temperature range 298–318 K.

**Fig. 5:**
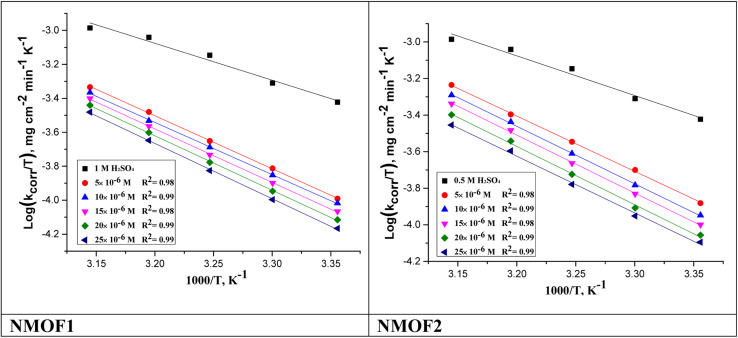
log(*k*_corr_/*T*) *vs.* 1/*T* of NMOF1 & NMOF2 with and without altered doses of MOFs at temperature range 298–318 K.

The activation energy 
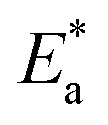
, which can be derived from eqn (3), is an essential component that influences the speed of reaction and the kind of adsorption.3
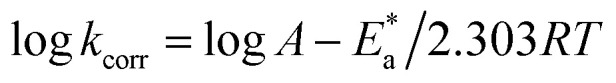
where *k*_corr_ is the corrosion rate, *A* is the Arrhenius constant, *R* is universal gas constant and *T* is the absolute temperature Fig. 4 depicts Arrhenius diagrams for NMOF1 and NMOF2 [log(*k*_corr_) *versus* 1/*T*], where the 
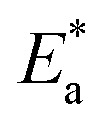
 energy of the analysis of the results was obtained in Table 4, It suggests that the surface reaction dominates the overall activity since the corrosion process 
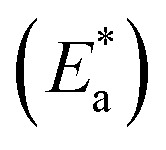
 is more than (20 kJ mol^−1^) and the activation energy increases as the dosage of metal–organic compound increases. Energy rises as the dose of metal–organic compound increases, it appears that the surface reaction dominates the overall activity. The adsorption nature of metal–organic compounds on C-steel causes this rise, which correlates to the physical adsorption of metal–organic compounds.^45–48^ The transitional state equation was used to calculate the changes in entropy and enthalpy. The activation enthalpy (ΔH*) and activation entropy (ΔS*) increase for C-steel corrosion in 0.5 M H_2_SO_4_ is calculated using the equation below:4
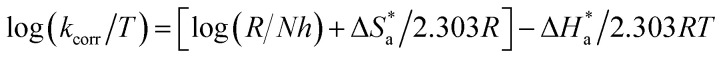
where symbol “*h*” is the Planck's constant and *N* is the Avogadro's number. Graph of log(*k*_corr_/*T*) *vs.* (1/*T*) for unprotected C-steel at 0.5 M H_2_SO_4_ and in the existence of metal–organic compounds is shown in Fig. 5, which gave straight lines with slope equal (−ΔH*/2.303*R*) and an intercept equal (log *R*/*Nh* − ΔS*/2.303*R*) from which ΔH* and ΔS* data were calculated and depicted in Table 4. Negative results of (ΔH*) on the C-steel surface, indicating that the reaction that occurs during the dissolving process is exothermic, and it is known that they may be used to chemical and physical adsorption.^49,50^ The mean values (ΔS*) are both high and negative, indicating that the activated complex is associated rather than dissociated during the rate-determining stage.

**Table 4:** C-steel dissolution parameters of (NMOF1 & NMOF2) with and without altered doses at 298–18 K

Comp.	Conc, ×10^6^ M	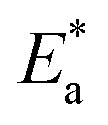 , kJ mol^−1^	ΔH*, kJ mol^−1^	−ΔS*, J mol^−1^ K^−1^
NMOF1	Blank	44.5 ± 0.2028	42 ± 0.2309	125 ± 0.2309
	5	63.6 ± 0.2403	61.1 ± 0.2027	71.1 ± 0.1732
	10	64.9 ± 0.2729	62.3 ± 0.1732	69.6 ± 0.2404
	15	65.5 ± 0.1453	62.9 ± 0.2603	68.7 ± 0.2028
	20	66.2 ± 0.1453	63.6 ± 0.2603	67.5 ± 0.2021
	25	67.0 ± 0.1856	64.5 ± 0.2333	65.9 ± 0.2048
NMOF2	5	60.6 ± 0.1528	58.0 ± 0.1787	80.2 ± 0.2603
	10	63.5 ± 0.1764	61.0 ± 0.1856	72.3 ± 0.1528
	15	64.2 ± 0.1856	61.6 ± 0.2048	71.5 ± 0.2603
	20	65.1 ± 0.1453	62.5 ± 0.1764	69.6 ± 0.1453
	25	66.1 ± 0.1856	63.5 ± 0.1856	67.8 ± 0.2048

#### 3.3.2. Adsorption isotherm behavior

**Fig. 6:**
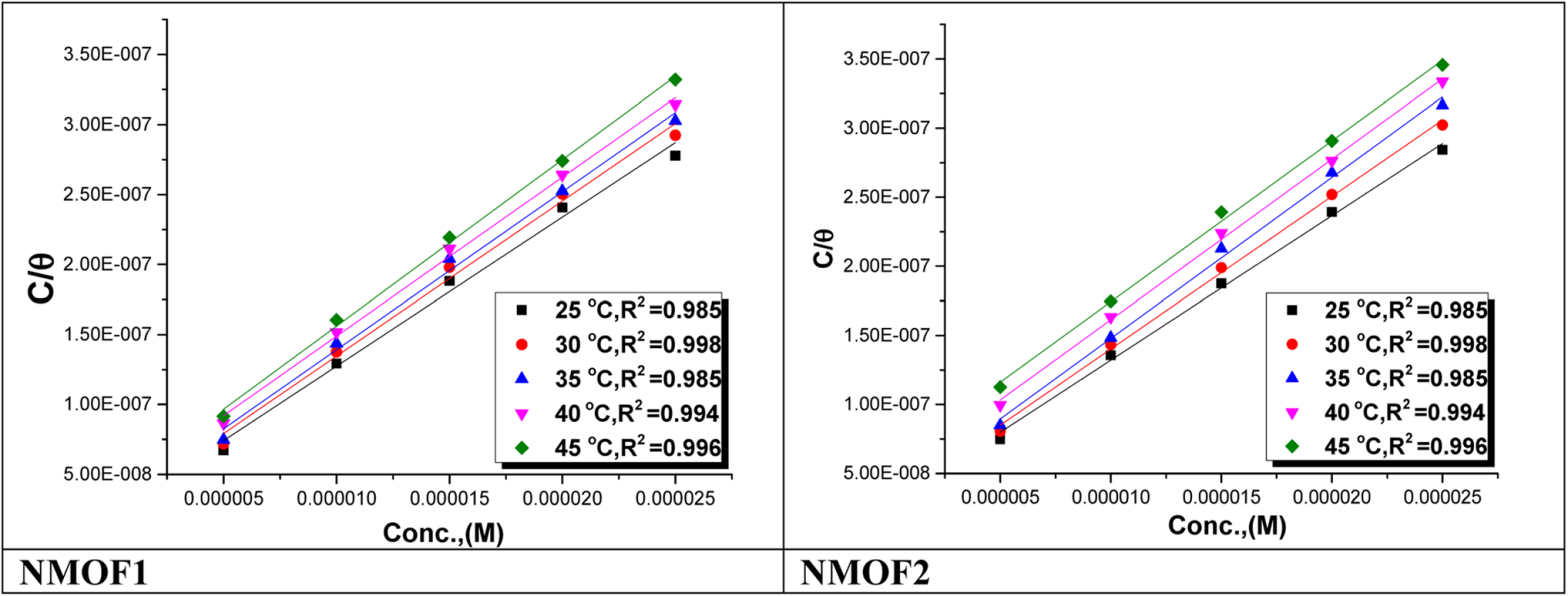
Langmuir isotherm of NMOF1 & NMOF2 at altered temperatures on a C-steel at 0.5 M H_2_SO_4_.

Studding of adsorption isotherms helps us to explain the reaction occurred among the C-steel surface and metal–organic additives. It is deduced that *θ* increased with raising the inhibitor dose; this is because of the adsorption of metal–organic additive molecules on the C-steel surface. It is also supposed that the adsorption of the studied metal–organic additives is proceeding with the monolayer adsorption so that the adsorption process may obey Langmuir isotherm. The *C*_inh_/relationship dependence for NMOF1 and NMOF2 is shown in Fig. 6, because of the dosage of metal–organic compounds (*C*_inh_) obeying Langmuir isotherm adsorption. According to the Langmuir isotherm, a metal surface has a specific number of adsorption sites for a single adsorbate, and Goads has a constant value for the sites regardless of the amount of surface covering.^51^

5 *C*/*θ* = 1/*K*_ads_ + *C*

where *K*_ads_ is the equilibrium adsorption constant intricate in chemical reaction6
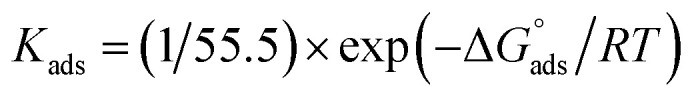
where 
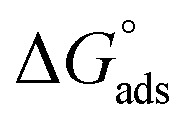
 is the standard free energy of adsorption, *C* is the concentration of the inhibitor in M, and 55.5 dosage of molar water in solution. The data pattern revealed a negative sign of 
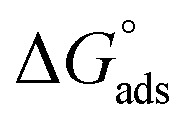
 due to the spontaneous and stable adsorbed layer on the metal surface.^52^ The adsorption characteristics for the metal–organic compounds found are shown in Table 5. The free energy findings show that the kind of adsorption event is physical (mainly) and chemical adsorption, since it is known that negative values are greater than 20 kJ mol^−1^ and less than 40 kJ mol^−1^ for the current study. Due to the nonhomogeneous character of the metal surface, including microscopic spaces, non-metallic inclusions, and impurities, the Goads values in Table 5 alter with changes in inhibitor concentration. This confirms the Langmuir model. The 
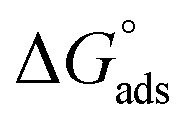
 values ranged between −22.7 and −23.1 kJ mol^−1^, suggesting physical and chemical adsorption (mixed adsorption). The enthalpy of adsorption, 
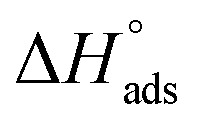
, was determined using the Vant Hoff equation:7
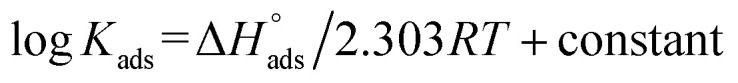


**Table 5:** Demonstrations the kinetic characteristics as a function of temperature for C-steel dissolution at 0.5 M H_2_SO_4_ in NMOF1 and NMOF2

Inhibitor	Temp., K	*K*_ads_, M^−1^	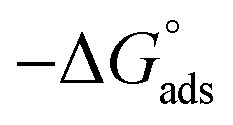 , kJ mol^−1^	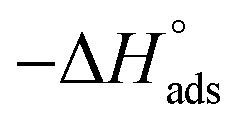 , kJ mol^−1^	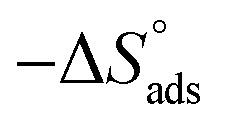 , J mol^−1^ K^−1^
NMOF1	298	48 ± 0.1859	19.5 ± 0.2025	37 ± 0.1645	57.3 ± 0.2033
	303	41 ± 0.1833	19.4 ± 0.2251		56.5 ± 0.2120
	308	34 ± 0.2141	19.3 ± 0.1654		56.1 ± 0.1654
	313	29 ± 0.2241	19.1 ± 0.1452		55.4 ± 0.1425
	318	22 ± 0.1741	18.8 ± 0.1325		55.1 ± 0.1345
NMOF2	298	41 ± 0.1525	19.2 ± 0.1253	33 ± 0.1841	47.3 ± 0.1228
	303	35 ± 0.2033	19.1 ± 0.1725		46.8 ± 0.2128
	308	30 ± 0.2236	19.0 ± 0.2121		46.2 ± 0.2014
	313	25 ± 0.2101	18.9 ± 0.2255		45.9 ± 0.2023
	318	22 ± 0.1788	18.8 ± 0.2125		45.5 ± 0.1745

**Fig. 7:**
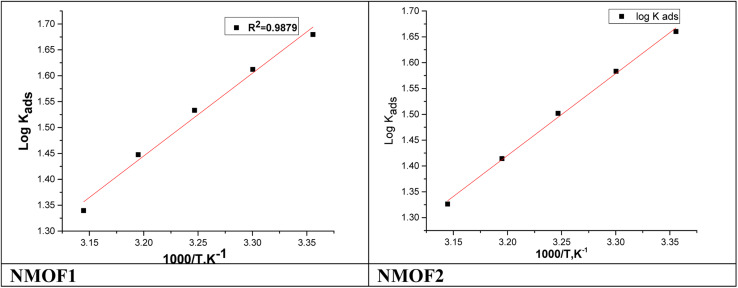
log *K*_ads_*vs. T* bends gotten from Langmuir isotherm for NMOF1 & NMOF2.

Fig. 7 shows plotting of log *K*_ads_ with 1/*T* for C-steel in 0.5 M H_2_SO_4_ with NMOF1 & NMOF2. The negative sign of the 
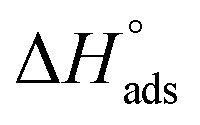
 data designates that the adsorption procedure is exothermic. Adsorption can be physical or chemical in an exothermic procedure, while it can only be chemical in an endothermic procedure. Finally, the next Eq. may be utilized to calculate 
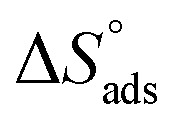
.8
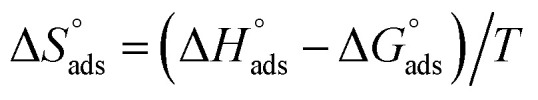


Table 5 demonstrations the values for 
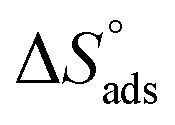
. The −ve sign of 
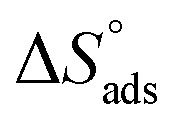
 values designates that the order of the adsorbed molecules at the solid/liquid contact is decreasing.

### 3.4. Electrochemical measurements

#### 3.4.1. PDP measurements

**Fig. 8:**
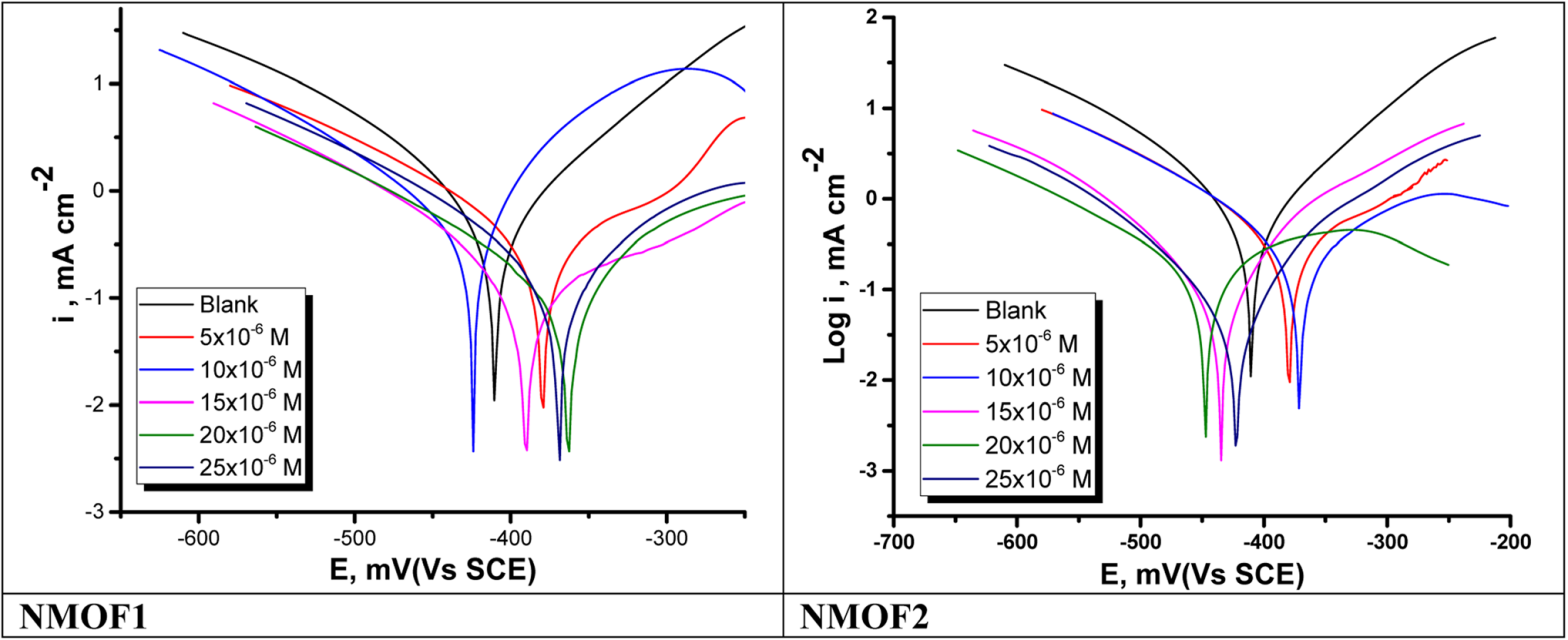
PDP bends for the liquefaction of C-steel in 0.5 M H_2_SO_4_ with and without altered doses of NMOF1 & NMOF2 at 298 K.

PDP diagrams of C-steel in 0.5 M sulfuric acid in the existence and absence of altered doses of metal–organic compounds at 298 K are shown in Fig. 8. From this figure we see that Tafel extrapolation obtained the electrochemical parameters at *E*_corr_ and were depicted in Table 6. The current density reduced as the accumulation of inhibitors increased. According to the results of the tests, *β*_c_ is somewhat greater than *β*_a_, suggesting that the inhibitors favor cathodic rather than anodic action. As a result, these inhibitors function like a combination of inhibitors. Also, *E*_corr_ change slightly (less than ±85 mV) which confirm that these compounds exert on both cathodic (hydrogen reduction) and anodic (metal dissolution) processes. The efficacy of inhibition (*η*%) was determined from the curves of polarization as in eqn (9):

9 *η*% = (1 − (*i*_corr_/*i*^o^_corr_))

where, *i*_corr_ and *i*^o^_corr_, respectively, are the current densities of corrosion with and without of metal–organic compounds (NMOF1 & NMOF2).^53,54^ The parallel Tafel lines with and without inhibitors indicate that there is no change in corrosion mechanism.

**Table 6:** Effect of NMOF1 & NMOF2 dose on (*E*_corr_), (*i*_corr_), (*β*_c_, *β*_a_), (*θ*) and (*η*%) of C-steel in 0.5 M H_2_SO_4_ at 298 K

[Inh]	Conc., ×10^6^ (M)	−*E*_corr_ mV *vs.* SCE	*i*_corr_, mA cm^−2^	*β*_a_, mV dec^−1^	−*β*_c_, mV dec^−1^	*θ*	*η*%
Blank	—	410.0 ± 0.1658	0.9327 ± 0.0183	267.0 ± 0.2028	145.0 ± 0.2028	—	—
NMOF1	5	379.6 ± 0.2033	0.5606 ± 0.0016	125.8 ± 0.1642	145.9 ± 0.1787	0.399	39.9
	10	424.1 ± 0.2444	0.4132 ± 0.0250	89.3 ± 0.1243	114.1 ± 0.1645	0.557	55.7
	15	390.3 ± 0.1547	0.3098 ± 0.0220	137.8 ± 0.2014	150.4 ± 0.1457	0.668	66.8
	20	363.1 ± 0.1754	0.1921 ± 0.0156	148.5 ± 0.2531	152.2 ± 0.1347	0.794	79.4
	25	369.4 ± 0.1562	0.1229 ± 0.0172	214.0 ± 0.2124	157.8 ± 0.1687	0.868	86.8
NMOF2	5	369.6 ± 0.1465	0.6043 ± 0.0152	135.7 ± 0.1655	151.9 ± 0.2134	0.352	35.2
	10	377.6 ± 0.1235	0.4719 ± 0.0132	124.3 ± 0.1892	143.7 ± 0.2245	0.494	49.4
	15	443.1 ± 0.1125	0.4019 ± 0.0186	148.8 ± 0.2121	129.8 ± 0.2217	0.569	56.9
	20	451.3 ± 0.2023	0.1977 ± 0.0521	119.4 ± 0.2301	159.6 ± 0.1787	0.788	78.8
	25	391.7 ± 0.2235	0.1343 ± 0.0148	114.3 ± 0.1871	160.5 ± 0.1975	0.856	85.6

#### 3.4.2. Electrochemical impedance spectroscopy (EIS) measurements

**Fig. 9:**
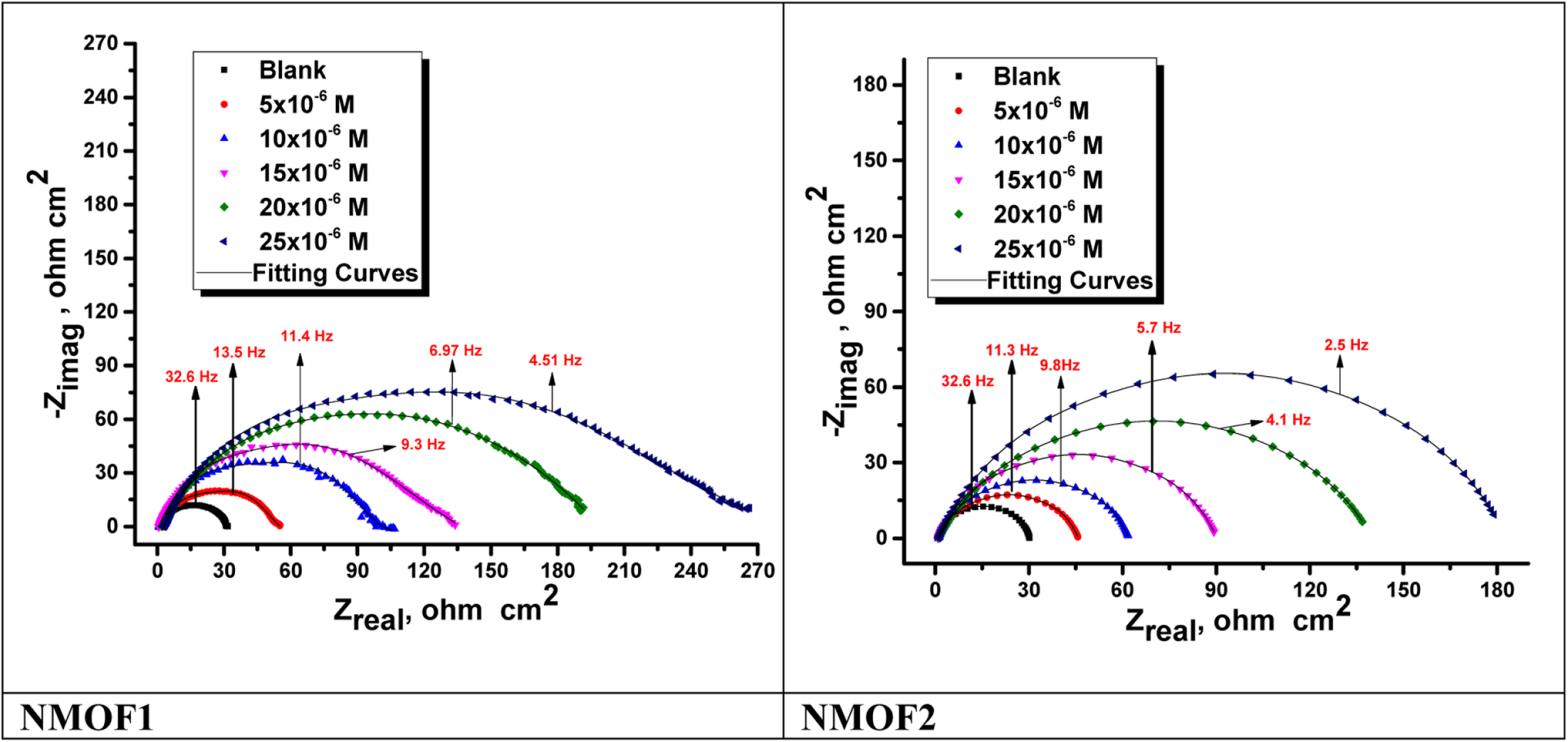
Nyquist plots for C-steel dissolving in 0.5 M H_2_SO_4_ in the presence and absence of altered doses of NMOF1 and NMOF2 at 298 K.

**Fig. 10:**
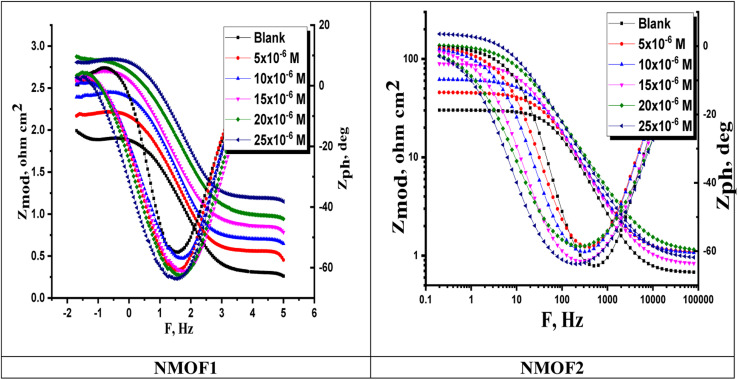
Bode graphs for C-steel dissolving in 0.5 M H_2_SO_4_ in the presence and absence of different doses of NMOF1 & NMOF2 at 298 K.

**Fig. 11:**
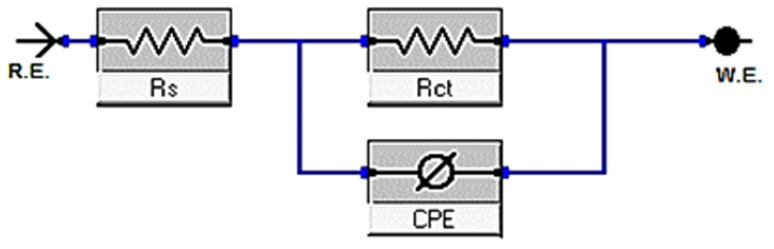
Equivalent circuit model used to fit experimental EIS.

Fig. 9 and 10 show the C-steel Nyquist and Bode diagrams at OCP in the absence and presence of different dosages of metal–organic MOF1 and MOF2 at 298 K. The circuit that represents metal organic compounds and electrolyte is presented in Fig. 11, with *R*_s_ as the solution resistance. The impedance spectra showed that the diameter increases as the dose of studied inhibitors rises. The interfacial capacitance *C*_dl_ values can be estimated from CPE parameters (*Y*_0_ and *n*) and is defined in eqn (10):^55–57^

10 *C*_dl_ = *Y*_0_(*ω*_max_)^*n*−1^

where, *Y*_0_ is the CPE magnitude, which is considered a surface irregularity of the electrode, causes a greater depression in Nyquist semicircle diagram, where the metal–solution interface acts as a capacitor with irregular surface, *ω*_max_ represents angular frequency, and *n* is the CPE component. If the electrode surface is homogeneous and plane, the exponential value of (*n*) = 1 and the metal-solution interface acts as capacitor with regular surface.^58^ The Bode graphs for the additives (Fig. 10) revealed that the single time constant stated in the Nyquist plot actually existed, which indicates that the corrosion of C-steel in 1 M HCl solution was governed by a single charge transfer process and that the addition of the additives to the system had no effect on the process.^59^Table 7 shows the impedance data that established the data of *R*_ct_ increasing with increasing the dosage of the metal–organic compounds, pointing to an increase in IE percent. This might be due to an increase in the thickness of the adsorbed layer caused by increasing the metal–organic compound dosages. Table 7 also shows that (*n*) value varies directly with NMOF1 and NMOF2 dosages. The value of (*n*) is a measure of surface roughness,^60^ and its rise might indicate a reduction in the heterogeneity of the metal surface caused by NMOF1 and NMOF2 adsorption. The inclusion of NMOF1 and NMOF2 results in lower *C*_dl_ values, which the Helmholtz model ascribed to an increase in the thickness of the electric double layer or/and a drop in the local dielectric constant:^61^

11 *C*_dl_ = *εε*°*A*/*δ*

where *ε* is the dielectric constant of the medium, *ε*° is vacuum permittivity, *A* is the electrode area and *δ* is the thickness of the protective layer. Bode graphs (Fig. 11) in the presence of inhibitors revealed that the Bode amplitude value increases over the whole frequency range with the addition of NMOF1 and NMOF2. Eqn (12) was used to get the percent IE and *θ* from the impedance testing:12
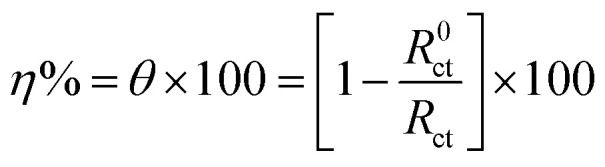
where, *R*^0^_ct_ and *R*_ct_ are the resistances unprotected and protected metal–organic compounds, individually. Table 7 shows the values of parameters such as *R*_s_ and *R*_ct_, *Y*_o_, *n* obtained from EIS fitting, as well as the derived parameters *C*_dl,_ Goodness of fit (*χ*^2^) and *η*%. Fig. 9 shows that the experimental and theoretical curves fit together nicely. The chi-squared test was used to gauge the accuracy of the fitting results, and the tiny chi-squared values (Table 7) obtained for each result indicate that the fitted results and experimental results agree very well.

**Table 7:** EIS parameters for the dissolving of C-steel in 0.5 M H_2_SO_4_ with and without changed dosages of studied metal–organic frameworks (NMOF1 & NMOF2) at 298 K

[Inh]	Conc. ×10^6^ M	*R*_s_, (Ω cm^2^)	*Y*_o_, μΩ^−1^ s^*n*^ cm^−2^	*n*	*C*_dl_, (μF cm^−2^)	*R*_ct_, (Ω cm^2^)	*η*%	Goodness of fit (*χ*^2^)
Blank	—	1.9283 ± 0.0183	689	0.960	586.9 ± 0.2321	30.4 ± 0.1757	—	16.88 × 10^−3^
NMOF1	5	2.1328 ± 0.0175	482	0.968	427.4 ± 0.1487	53.7 ± 0.2128	43.5	21.45 × 10^−3^
	10	2.4726 ± 0.0213	398	0.970	360.4 ± 0.1657	99.2 ± 0.1612	69.4	21.58 × 10^−3^
	15	2.670 ± 0.0230	303	0.973	276.1 ± 0.2587	128.1 ± 0.2109	76.3	19.10 × 10^−3^
	20	2.786 ± 0.0165	243	0.976	225.81 ± 0.1458	194.8 ± 0.2228	84.4	17.22 × 10^−3^
	25	2.791 ± 0.0244	176	0.978	164.42 ± 0.2102	260.6 ± 0.1932	88.4	17.33 × 10^−3^
NMOF2	5	1.882 ± 0.0147	499	0.979	460.1 ± 0.1753	47.2 ± 0.1828	35.7	20.11 × 10^−3^
	10	1.954 ± 0.0214	437	0.983	410.5 ± 0.1987	61.8 ± 0.1744	50.9	21.16 × 10^−3^
	15	2.232 ± 0.0145	393	0.985	373.3 ± 0.2112	89.4 ± 0.2121	66.0	14.87 × 10^−3^
	20	2.336 ± 0.0157	261	0.987	249.7 ± 0.1722	138.7 ± 0.1857	78.1	16.75 × 10^−3^
	25	2.449 ± 0.0168	242	0.989	233.4 ± 0.1653	181.1 ± 0.1732	83.2	17.5810^−3^

### 3.5. Surface analysis by FT-IR analysis

**Fig. 12:**
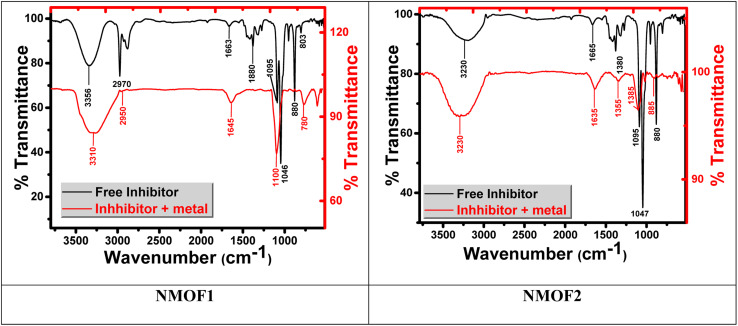
(a) FTIR spectra for free, and (b) FTIR spectra of metal with NMOF1 & NMOF2.

Fourier transform infrared spectroscopy (FT-IR) identifies chemical bonds in a molecule by producing an infrared absorption spectrum. FT-IR spectrum of the corrosion product at C-steel surface in 0.5 M H_2_SO_4_ does not show any useful adsorption peaks.^62,63^ FT-IR fingerprint spectra of the stock metal–organic EIS and the C-steel surface after dipping in 0.5 M H_2_SO_4_ + 25 × 10^−6^ M of metal–organic NMOF1 for 24 hours was obtained and compared to each other it was obviously clear that the same fingerprint of metal–organic. NMOF1 solution present on C-steel surface except the absence of some functional group and it suggested being due to reaction with H_2_SO_4_. From Fig. 12 there are small shift in the peaks at C-steel surface from the original peak of the stock inhibitor solution, these shifts indicate that there is interaction between C-steel NMOF1 & NMOF2.

### 3.6. Corrosion inhibition mechanism

The inhibitory process in acid media begins with adsorption on the metal surface.^64^ Most studies on inhibition hypothesize that the unoccupied d-orbitals of the metal and the inhibitor's p-electrons create a donor–acceptor surface complex.^65^ The synthetic compounds for metal organic frameworks have a lot of surface area that is easily adsorbable to the metal surface to create a corrosion-inhibiting coating. Moreover, when coordinated with metal ions, the nitrogen and oxygen in the metal organic framework molecule are frequently positively charged and capable of drawing the chloride ions. To inhibit corrosion, lower the amount of chloride ions present on the metal surface.^66^ In aqueous acidic solutions, the inhibitor is either neutral or takes the form of cations (*i.e.*, protonated species). Two different adsorption kinds could be generally thought of. As a result of the displacement of water molecules from the metal surface and the sharing of electrons between N, O atoms and Fe as well as between the π-electrons of the aromatic ring and the open d-orbitals of Fe, the neutral form of the inhibitor may adsorb on the metal surface. On the other hand, it is well known that the steel surface is positively charged under acidic circumstances.^67^ As a result, a protonated inhibitor has suffering adhering to the positively charged steel surface due to electrostatic repulsion. Because they are more strongly adsorbed SO_4_^2+^ ions produce an excess negative charge in the solution, which encourages the adsorption of more protonated inhibitors. When the protonated inhibitor is deposited on the metal surface, two things could happen: (i) a coordinate bond could form as a result of a partial electron transfer from N atoms to the metal surface, and (ii) the protonated inhibitor could combine with recently formed Fe^2+^ ions to form a metal-inhibitor complex on the surface of C-steel.^67^Fe^2+^ + *x* inh → [inh_*x*_ + Fe]^(2+*x*)+^

These complexes may be adsorbed on the steel surface by van der Waals forces, generating a corrosion-resistant covering. The film blocks both cathodic and anodic processes at the same time.^68^ As a result, the primary mechanism of the inhibitor's corrosion inhibition is the adsorption of inhibitor molecules containing N heteroatoms and π-electrons of aromatic rings on the surface of steel.

## 4. Conclusions

From the overall experimental results, the following conclusions can be deduced:

(1) Organometallic framework compounds NMOF1 & NMOF2 were synthesized and characterized by altered techniques.

(2) The investigated NMOFs compounds are excellent corrosion inhibitors for C-steel in 0.5 M H_2_SO_4_ solution.

(3) The effect acquired from all measurements confirmed that the inhibiting action will increase with elevating the NMOFs concentration and reduces with the increasing the temperature.

(4) All measured methods results showed that the inhibition performance of NMOF1 was better than that of NMOF2, and the inhibition rate of NMOF1 was 90.0%. This may be because NMOF1 was a large molecule complex, which can effectively form a cover layer, thereby enabling better corrosion inhibition of steel sheets.

(5) Double layer capacitance (*C*_dl_) lowered while *R*_ct_ increases with improving the concentration of NMOFs. This reality confirms the adsorption of these molecules at the C-steel surface obeying Langmuir adsorption isotherm. The adsorption constants *K*_ads_ of NMOF1 and NMOF2 were 48 ± 0.1859 L mg^−1^ and 41 ± 0.1515 L mg^−1^, respectively, and the inhibition ability of NMOF1 was better than that of NMOF2.

(6) PDP data revealed that these NMOFs performance as mixed type inhibitors.

(7) The negative values of 
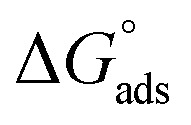
 indicate the spontaneity and stability of the adsorbed layer of the NMOFs on the surface of C-steel.

(8) The *η*% values gotten from the altered independent quantitative techniques show the validity of the results.

## Data availability

The authors confirm that the data supporting the findings of this study are available within the article and/or its ESI.†

## Author contributions

S. E. H and A. S. F. analyzed all measurement data and then wrote the manuscript. G. S. H and A. A. E. designed the experiments. All authors discussed the results and revised the manuscript.

## Conflicts of interest

The authors declare that we have no competing financial interests or personal relationships that could have appeared to influence the work reported in this paper.

## Supplementary Material

RA-013-D3RA01644G-s001

## References

[cit1] Nwokolo I. K., Shi H., Ikeuba A. I., Gao N., Li J., Ahmed S., Liu F. (2022). Coatings.

[cit2] Tang F., Chen G., Brow R. K., Koenigstein M. L. (2014). Materials.

[cit3] Fouda A. S., Ismail M. A., Khaled M. A., El-Hossiany A. A. (2022). Sci. Rep..

[cit4] Liu S., Xu N., Duan J., Zeng Z., Feng Z., Xiao R. (2009). Corros. Sci..

[cit5] Lowmunkhong P., Ungthararak D., Sutthivaiyakit P. (2010). Corros. Sci..

[cit6] Obot I. B., Obi-Egbedi N. O. (2010). Corros. Sci..

[cit7] Ahamad I., Prasad R., Quraishi M. A. (2010). Corros. Sci..

[cit8] Soltani N., Behpour M., Ghoreishi S. M., Naeimi H. (2010). Corros. Sci..

[cit9] Nikolova R. D., Vayssilov G. N., Rodios N., Bojilova A. (2002). Molecules.

[cit10] El-Agrody A. M., El-Latif A., El-Hady N. A., Fakery A. H., Bedair A. H. (2001). Molecules.

[cit11] Mahdavian M., Ashhari S. (2010). Electrochim. Acta.

[cit12] El-Saghier A. M. M., Khodairy A. (2000). Phosphorus, Sulfur Silicon Relat. Elem..

[cit13] Kovalenko S. M., Bylov I. E., Sytnik K. M., Chernykh V. P., V Bilokin Y. (2000). Molecules.

[cit14] Flašík R., Stankovičová H., Gáplovský A., Donovalová J. (2009). Molecules.

[cit15] Čačić M., Pavić V., Molnar M., Šarkanj B., Has-Schön E. (2014). Molecules.

[cit16] Azizian J., Mohammadi A. A., Bidar I., Mirzaei P. (2008). Monatsh. Chem..

[cit17] V Satyanarayana V. S., Sreevani P., Sivakumar A., Vijayakumar V. (2008). Arkivoc.

[cit18] Garazd M. M., V Muzychka O., Vovk A. I., V Nagorichna I., Ogorodniichuk A. S. (2007). Chem. Nat. Compd..

[cit19] Smitha G., Sanjeeva Reddy C. (2004). Synth. Commun..

[cit20] Kotali A., Lafazanis I. S., Harris P. A. (2008). Synth. Commun..

[cit21] Nofal Z. M., El-Zahar M. I., El-Karim A. (2000). Molecules.

[cit22] Piller N. , in Coumarins: Biology, Applications and Mode of Action, John Wiley & Sons Ltd, 1997, pp. 185–208

[cit23] Zahradník M. , The production and application of fluorescent brightening agents, Wiley, 1982

[cit24] Heravi M. M., Sadjadi S., Oskooie H. A., Shoar R. H., Bamoharram F. F. (2008). Catal. Commun..

[cit25] Soliman S. A., Metwally M. S., Selim S. R., Bedair M. A., Abbas M. A. (2014). J. Ind. Eng. Chem..

[cit26] Kumaraguru S., Pavulraj R., Mohan S. (2017). Trans. IMF.

[cit27] Zhang M., Ma L., Wang L., Sun Y., Liu Y. (2018). ACS Appl. Mater. Interfaces.

[cit28] Duan S., Dou B., Lin X., Zhao S., Emori W., Pan J., Hu H., Xiao H. (2021). Colloids Surf., A.

[cit29] Dehghani A., Poshtiban F., Bahlakeh G., Ramezanzadeh B. (2020). Constr. Build. Mater..

[cit30] Fouda A. S., Etaiw S. E. H., Hassan G. S. (2021). Sci. Rep..

[cit31] Alshima'a A. M., Hefnawy A., Langer V., Khatab M. A., Öhrstrom L., Abu-Youssef M. A. M. (2009). Polyhedron.

[cit32] Fouda A. S., Etaiw S. E.-D. H., El-bendary M. M., Maher M. M. (2016). J. Mol. Liq..

[cit33] Etaiw S. E. H., Fouda A. S., El-bendary M. M. (2013). Prot. Met. Phys. Chem. Surf..

[cit34] Nwokolo I. K., Shi H., Ikeuba A., Gao N., Li J., Ahmed S., Liu F. (2022). Coatings.

[cit35] Etaiw S. E. H., Fouda A. S., Amer S. A., El-bendary M. M. (2011). J. Inorg. Organomet. Polym. Mater..

[cit36] Khaled M. A., Ismail M. A., El-Hossiany A. A., Fouda A. S. (2021). RSC Adv..

[cit37] Etaiw S. E. H., El-bendary M. M., Fouda A. S., Maher M. M. (2017). Prot. Met. Phys. Chem. Surf..

[cit38] Zafari S., Shahrak M. N., Ghahramaninezhad M. (2020). Met. Biomater. Interfaces.

[cit39] Etaiw S. E. H., Marie H., Elsharqawy F. A. (2020). Appl. Organomet. Chem..

[cit40] Altomare A., Cascarano G., Giacovazzo C., Guagliardi A., Burla M. C., t Polidori G., Camalli M. (1994). J. Appl. Crystallogr..

[cit41] Asan A., Soylu S., Kıyak T., Yıldırım F., Öztaş S. G., Ancın N., Kabasakaloğlu M. (2006). Corros. Sci..

[cit42] Hanika-Heidl H., Etaiw S. E. H., Ibrahim M. S., El-din A. S. B., Fischer R. D. (2003). J. Organomet. Chem..

[cit43] Etaiw S. E. H., Abdou S. N. A. (2010). J. Inorg. Organomet. Polym. Mater..

[cit44] Salem A. M., Wahba A. M., El Hossiany A., Fouda A. S. (2022). J. Indian Chem. Soc..

[cit45] Tirbonod F., Fiaud C. (1978). Corros. Sci..

[cit46] Chu D. T. W., Claiborne A. K., Clement J. J., Plattner J. J. (1992). Can. J. Chem..

[cit47] Frigola J., Pares J., Corbera J., Vano D., Merce R., Torrens A., Mas J., Valenti E. (1993). J. Med. Chem..

[cit48] Barlin G. B., Nguyen T. M. T., Kotecka B., Rieckmann K. H. (1992). Aust. J. Chem..

[cit49] Al-Amiery A. A., Kadhum A. A. H., Kadihum A., Mohamad A. B., How C. K., Junaedi S. (2014). Materials.

[cit50] Mohamad A. B., Kadhum A. A. H., Al-Amiery A. A., Ying L. C., Musa A. Y. (2014). Met. Biomater. Interfaces.

[cit51] Eddy N. O., Mamza P. A. P. (2009). Port. Electrochim. Acta.

[cit52] Dakhil R. M., Gaaz T. S., Al-Amiery A. A., Kadhum A. A. H. (2018). Green Chem. Lett. Rev..

[cit53] Banerjee G., Malhotra S. N. (1992). Corrosion.

[cit54] Abd El-Maksoud S. A. (2002). Corros. Sci..

[cit55] Fouda A. S., Abd El-Maksoud S. A., Belal A. A. M., El-Hossiany A., Ibrahium A. (2018). Int. J. Electrochem. Sci..

[cit56] Abdel-Wahab B. F., Mohamed H. A., Farhat A. A. (2014). Org. Commun..

[cit57] Leleu S., Rives B., Caussé N., Pébère N. (2019). J. Magnesium Alloys.

[cit58] Behpour M., Ghoreishi S. M., Soltani N., Salavati-Niasari M., Hamadanian M., Gandomi A. (2008). Corros. Sci..

[cit59] Fouda A. S., El-Mekabaty A., Shaaban I. E. I., El-Hossiany A. (2021). Prot. Met. Phys. Chem. Surf..

[cit60] Fouda A. S., El-Maksoud S. A. A., El-Hossiany A., Ibrahim A. (2019). Int. J. Electrochem. Sci..

[cit61] Dewar M. J. S., Storch D. M. (1985). J. Am. Chem. Soc..

[cit62] Noor E. A., Al-Moubaraki A. H. (2008). Mater. Chem. Phys..

[cit63] Koopmans T. (1933). Physica.

[cit64] Rahiman A. F. S. A., Sethumanickam S. (2017). Arabian J. Chem..

[cit65] Tan B., He J., Zhang S., Xu C., Chen S., Liu H., Li W. (2021). J. Colloid Interface Sci..

[cit66] Obot I. B., Gasem Z. M., Umoren S. A. (2014). Int. J. Electrochem. Sci..

[cit67] Li X., Deng S., Li N., Xie X. (2017). J. Mater. Res. Technol..

[cit68] Ge L., Zuo F., Liu J., Ma Q., Wang C., Sun D., Bartels L., Feng P. (2012). J. Phys. Chem. C.

