# Supplementary material for: Nano-metal–organic frameworks as corrosion inhibitors for strengthening anti-corrosion behavior of carbon steel in a sulfuric acid environment: from synthesis to applications
Source: RSC Adv. 2023 May 18;13(22):15222–35. doi: 10.1039/d3ra01644g (PMC10193202; doi:10.1039/d3ra01644g)
Supplement: RA-013-D3RA01644G-s001 [file RA-013-D3RA01644G-s001.pdf]

Electronic Supplementary Material (ESI) for RSC Advances.
This journal is © The Royal Society of Chemistry 2023

Nano-metal–organic frameworks as corrosion inhibitors for strengthening anti-corrosion behavior of carbon steel in sulfuric acid environment: From synthesis to applications

Supplementary Materials

Table SF1 Bond lengths (Å) and bond angles (deg.) of NMOF**1**

N9- Cu8	1.866	Cu8- N7- C8	170.24
N7- Cu8	1.879	N9- C10- Cu11	137.36
Cu24- C25	1.999	N2- C1- Cu24	177.06
N21- Cu24	2.256	C1- N2- Sn3	149.23
Cu11- C29	2.008	Sn3- C4- N5	177.40
Cu11- N15	2.267	C10- Cu11- N15	107.59
C10- Cu11	2.025	C10- Cu11- N28	117.66
C6- Cu24	2.032	C10- Cu11- C29	107.00
C1- Cu24	2.015	N15- Cu11- C22	104.41
Sn3- Cu7	2.270	N28- Cu11- C29	113.93
Sn3- C37	2.277	C1- Cu24- N21	103.18
Sn3- C34	2.302	C1- Cu24- C25	111.75
Sn3- C4	2.313	C8- Cu24- N21	108.69
Sn3- N2	2.773	C1- Cu24- C6	112.83
C1- N2	1.209	C6- Cu24- N2	108.00
C6- N7	1.214	N2- Sn3- C37	89.83
N9- C10	1.208	N2- Sn3- C34	80.90
C4- N5	1.211	C4- Sn3- C47	93.01
C27- N28	1.208	C4- Sn3- C37	89.13
C29- N30	1.208	C34- Sn3- C47	124.94
C52-O53	1.273	C37- Sn3- C47	103.70
C49- O50	1.268	C34- Sn3- C37	128.27

Table SF2 Selected bond lengths (Å) and bond angles (°) of NMOF2.

C1—Cu1	1.9215(13)	N2—C2—Cu1	176.78(11)
C1—N1	1.1566(17)	C2—Cu1—C1	125.91(6)
C2—N2	1.1569(16)	C2—Cu1—N3	110.20(5)
C2—Cu1	1.9243(12)	C1—Cu1—N3	103.16(5)
C3—Sn1	2.1201(13)	C2—Cu1—N4	102.48(5)
C4—Sn1	2.1210(13)	C1—Cu1—N4	113.04(5)
C5 —Sn1	2.1136(13)	N3—Cu1—N4	98.99(5)
Cu1—N3	2.1365(11)	C1—N1—Sn1	176.61(12)
Cu1—N4	2.1259(11)	C2—N2—Sn1	175.46(11)
N1—Sn1	2.3229(12)	N1—Sn1—N2	178.98(4)
N2—Sn1	2.3128(12)	N2—Sn1—C4	90.79(5)
Cu1—C1—N1	175.89(12)	N1—Sn1—C3	89.93(6)
N2—Sn1—C3	90.46(6)	N2—Sn1—C5	88.61(5)
C4—Sn1—C3	118.64(6)	C4—Sn1—C5	120.32(6)
N1—Sn1—C5	90.38(5)	C3—Sn1—C5	121.04(6)
Symmetry codes: (i) *x*, -*y*+3/2, *z*+1/2; (ii) -*x*+2, -*y*, -*z*; (iii) -*x*+1, -*y*, -*z*; (iv) *x*, -*y*+3/2, *z*-1/2			

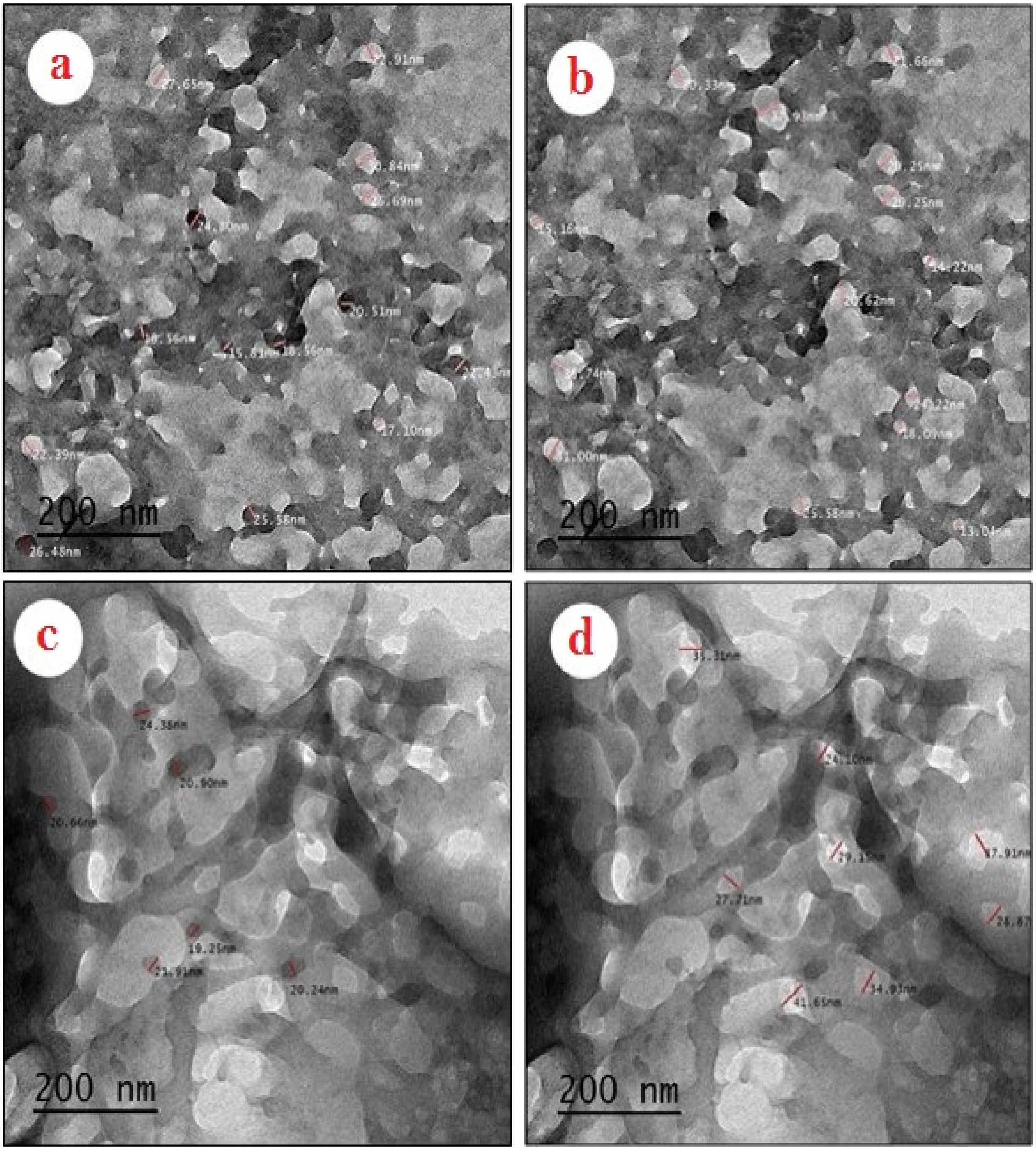

Figure SF1 TEM images of NMOF1 (a and b) and of NMOF2 (c and d)
